# Controlling amyloid formation of intrinsically disordered proteins and peptides: Slowing down or speeding up?

**DOI:** 10.1042/EBC20220046

**Published:** 2022-12-16

**Authors:** Yong Xu, Roberto Maya-Martinez, Sheena E. Radford

**Affiliations:** 1 Astbury Centre for Structural Molecular Biology, School of Molecular and Cellular Biology, Faculty of Biological Sciences, University of Leeds, LS2 9JT, United Kingdom

## Abstract

The pathological assembly of intrinsically disordered proteins/peptides (IDPs) into amyloid fibrils is associated with a range of human pathologies, including neurodegeneration, metabolic diseases and systemic amyloidosis. These debilitating disorders affect hundreds of millions of people worldwide and the number of people affected is increasing sharply. However, the discovery of therapeutic agents has been immensely challenging largely because of i) the diverse number of aggregation pathways and the multi-conformational and transient nature of the related proteins or peptides and ii) the under-development of experimental pipelines for the identification of disease-modifying molecules and their mode-of-action. Here, we describe current approaches used in the search for small-molecule modulators able to control or arrest amyloid formation commencing from IDPs and review recently reported accelerators and inhibitors of amyloid formation for this class of proteins. We compare their targets, mode-of-action and effects on amyloid-associated cytotoxicity. Recent successes in the control of IDP-associated amyloid formation using small molecules highlight exciting possibilities for future intervention in protein-misfolding diseases, despite the challenges of targeting these highly dynamic precursors of amyloid assembly.

## Introduction

Amyloidosis is a class of protein-misfolding diseases characterised by the deposition of amyloid fibrils in the tissues or organs of the individuals affected [1, 2]. The most prevalent amyloid disorders, which affect hundreds of millions of individuals worldwide include neurodegenerative diseases (Alzheimer’s disease (AD), Parkinson’s disease (PD), and Amyotrophic Lateral Sclerosis (ALS)). Amyloidosis can also involve systemic or localised amyloid deposits, and includes disorders such as Dialysis-Related Amyloidosis (DRA) immunoglobulin light chain (AL) amyloidosis) and metabolic disease-related (type II diabetes (T2D)). The formation of amyloid involves the aberrant self-association of one or more proteins or peptides into the cross-β structure canonical of amyloid fibrils [3]. While more than 50 human proteins are currently known to be the precursors of human amyloid disease [1, 2], ∼30% of these precursors are intrinsically disordered proteins/peptides (IDPs), which are able to adopt wide variety of conformations in their functional monomeric states. Two types of the most widely studied amyloid precursors are dynamically unstructured peptides: the amyloid β (Aβ) peptides (Aβ_40_ and Aβ_42_) [2, 4] and 37-residue human islet amyloid peptide (hIAPP/amylin) [5] which constitute the main amyloid components in plaques detected in people with AD and T2D, respectively. Of particular interest is the increasing recognition of the potential link between T2D and neurodegenerative disorders. Not only have *in vitro* studies shown the cross-assembly of Aβ and hIAPP [6–8], but mixed amyloid deposits of hIAPP and Aβ have also been detected in AD [9, 10]. These findings raised the proposal of a new neuroendocrine disorder referred to as type 3 diabetes [11, 12].

Recent cryo-electron microscopy (cryo-EM) studies have revealed atomic resolution insights into the structure of amyloid fibrils, with the surprising result that the end products of these self-association processes can be polymorphic, yet specific to each disease kind [3, 13]. Kinetic analysis has shown that the self-assembly of amyloidogenic precursors generally occurs via a nucleation-dependent mechanism, involving the formation of a broad range of conformational ensembles of monomeric and multimeric species [1, 14]. However, the exact mechanism of fibril formation remains unclear, especially regarding the structures of the different species involved and the nature of the different kinetic steps of primary nucleation, secondary nucleation and elongation. As a consequence, routes to intervention in amyloid disease by structure-based design of small molecules is a difficult task [15–18]. An additional complexity in the design of therapies against amyloidosis is the current lack of understanding of the culprit species of the cytotoxicity associated with amyloid formation [16, 19–21], which is partly due to the lack of chemical tools to purposely manipulate amyloidogenic systems, such as stabilising the original transient and dynamic species and limiting the heterogeneity of the aggregation processes. Development of such small-molecule modulators would not only enable us to better understand the mechanisms of aggregation into amyloid and its associated cytotoxicity, but would also provide us a clearer answer to the question of whether it is better to slow down or speed up amyloid formation as a therapeutic strategy.

Here we review current strategies for the discovery of small-molecule modulators against different IDPs involved in amyloid diseases. We then summarise recently reported small-molecule modulators (both inhibitors and accelerators) of the self-assembly of IDPs, mainly focussing on hIAPP and Aβ. We describe current biochemical and biophysical techniques that can be used to define the targets and mode-of-action of these interactions and summarise future challenges and possible solutions to the important question of how to better understand and treat amyloid diseases using small molecules.

### The challenges in controlling the aggregation kinetics of IDPs using small molecules

Many protein-based modulators of amyloid formation of various IDPs have been reported, including antibodies, nanobodies and molecular chaperones [22–25], but we still lack effective chemical tools able to purposely manipulate an aggregation energy landscape so as to limit the intermediates formed and/or to control the products of assembly (fibril structures or other aggregation types [26]). Consequently structure-function relationships traditionally used to understand biological mechanisms cannot be carried out. IDPs are extremely challenging to target using small molecules as: 1) they are intrinsically disordered, hence lack structured binding sites; 2) oligomeric intermediates of amyloid assembly are heterogeneous and transient; 3) the toxic species in amyloid assembly remain elusive, such that we currently lack defined species to target. Moreover, protein-misfolding diseases are under kinetic control. Hence, instead of designing chemical tools which target structured protein pockets driven by thermodynamic control, the primary strategy for ligand design lies in the modulation of the aggregation kinetics by entropy-driven binding and the formation of transient and weak interactions between small molecules and IDPs [27–30].

**Fig. 1:**
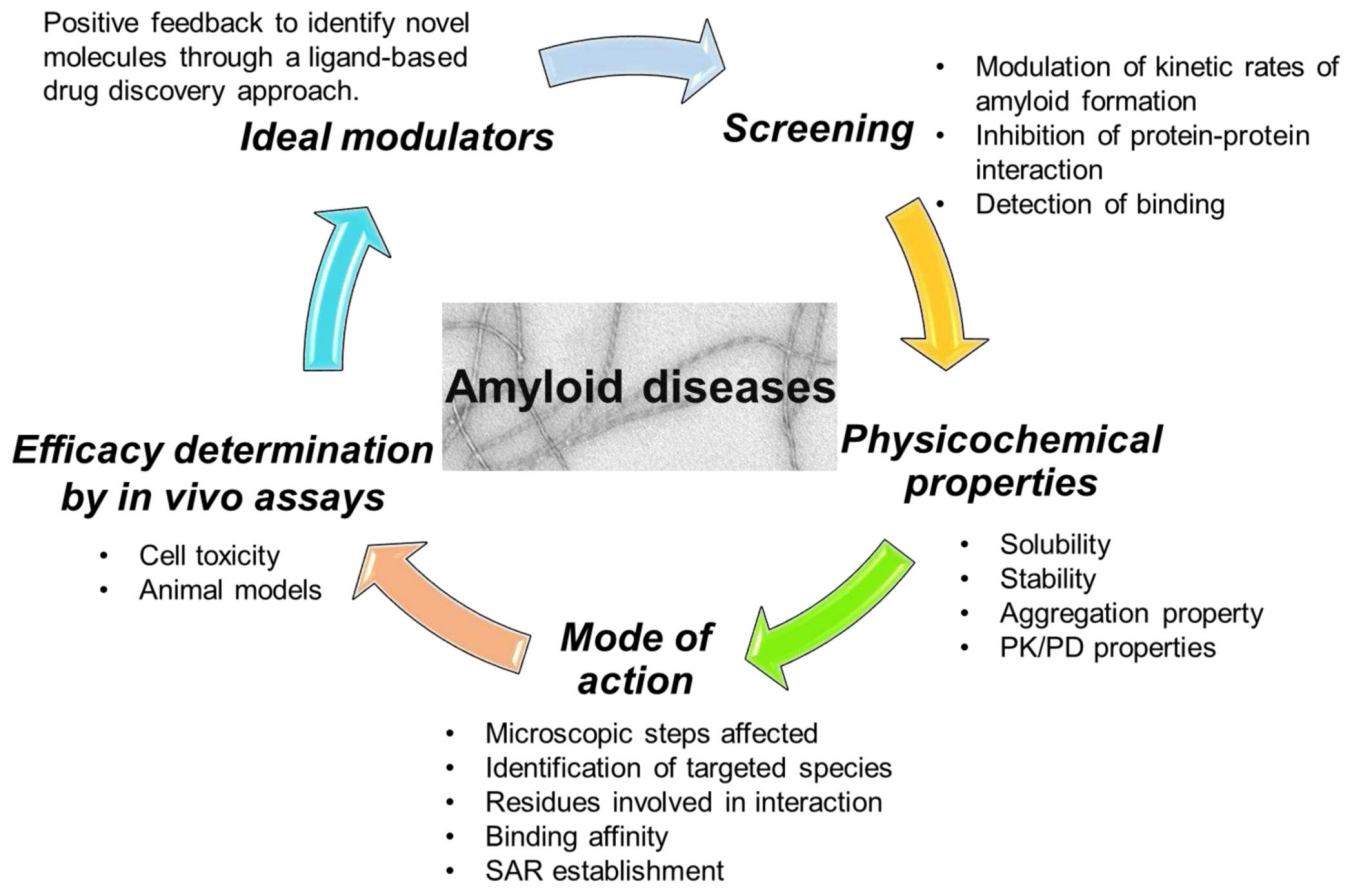
Linked steps in a campaign of modulator discovery towards IDPs involved in protein aggregation into amyloid. The order of the steps for modulator discovery does not matter, as all are needed for successful modulator discovery. PK (Pharmacokinetics), PD (Pharmacodynamics) and SAR (Structure-Activity Relationship).

In general, there are five crucial steps for a successful campaign of modulator discovery towards IDPs (Fig. 1). Compared to conventional drug discovery programs, screening for amyloid modulators starts with the lack of defined targets or detailed structural information. Consequently, many studies have relied on *in vitro* or *in vivo* biochemical screening of compound libraries, using assays that monitor fibril formation (famously using thioflavin T (ThT) *in vitro* [31, 32]), directly detecting binding events (e.g. using native electrospray ionisation mass spectrometry (nESI-MS) [33, 34] or using cellular screening [35, 36]). The next crucial stage involves probing the mode-of-action of these “hits”, including identifying the species which bind the small molecules, where the small molecules bind, how strong the interaction is, and the effect(s) of the small molecules on the different steps and the mechanism of amyloid formation. Efficacy *in vivo*, including evaluation the effect of the small molecules in cellular assays or animal models provides another crucial hurdle. All have to be understood to define the mode-of-action of the small molecule in order to take it forwards for exploration of its effect in an organismal setting.

### Methods to search for modulators against the aggregation of IDPs into amyloid

**Fig. 2:**
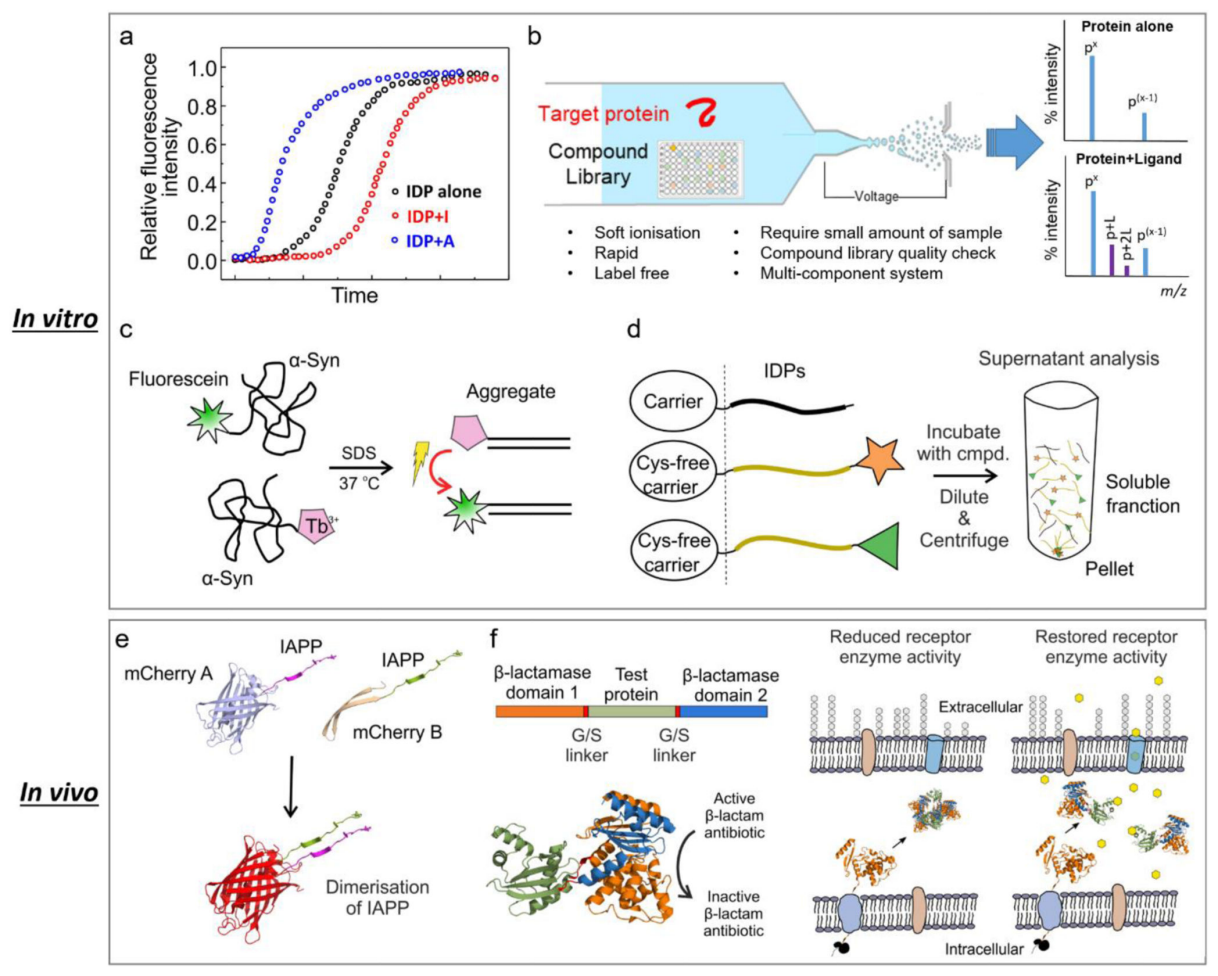
Different approaches have been applied for the discovery of small-molecule modulators against IDPs. (a) Fluorescence-based ThT assays. These assays are usually practiced in miniaturised formats with low cost. However, they require extremely pure proteins/peptides to achieve kinetic data with high reproducibility. Also the assay conditions (such as buffer, temperature, shaking or non-shaking, etc.) need to be carefully controlled. (b) Screening by nESI-MS. Under soft ionisation conditions, the non-covalent interactions between protein and ligand can be maintained. nESI-MS is label-free, can be used in multi-component systems, and confirms the integrity of the compound library at the same time. However caution should be exercised due to the inherent drawbacks of nESI-MS, such as non-specific binding during the electrospray process. (c) FRET-based assays have been developed for HTS of large library to identify small-molecule modulators *in vitro*. This figure was adapted from ref [46]. (d) Synergistic Aggregation Modulator Assay (SynAggreg) is an *in vitro* HT platform for the study of protein aggregation and the effect of modulators on protein aggregation [47]. This figure was adapted from ref [47]. (e) A bimolecular fluorescence complementary assay (BiFC) in a constructed *E. coli* system was developed to monitor the initial transient dimerization stage [35]. Specifically, mCherry protein was split and fused into amyloidogenic peptides/proteins, and a strong fluorescent signal can be detected if the fused biomolecules self-assemble into dimers. This figure was adapted from ref [35]. (f) β-Lactamase tripartite fusion system in *E. coli* has been introduced to screen inhibitors that prevent protein aggregation [36]. The bioassay can be configured in a 48-well format and has a simple phenotypic antibiotic resistance readout which directly links to the aggregation events of the test proteins/peptides. This figure was adapted from ref [36]. *In vivo* screening using different organisms have also been reported, such as *C. elegans* [48, 49]. One of the most important features of these systems is that they are able to investigate the amyloid-forming protein/peptide induced toxicity including those transiently populated low molecular weight oligomers.

Any of the many steps involved in an amyloid formation are a potential target for small-molecule modifiers of amyloid formation. Some of the approaches developed for conventional drug discovery, as well as newly established approaches specifically targeting IDPs, have been applied in the search for new chemical entities able to modulate amyloidogenesis. These approaches can be broadly grouped into four strategies:1) high-throughput screening (*in vitro* and *in vivo*) [31, 33, 36–40]; 2) structure-based drug design (mostly targeting amyloid fibrils) [41, 42]; 3) sequence-based modulator discovery (mostly peptide modulators) [43]; and 4) fragment-based approaches [44]. Despite the activity in this field, only one small molecule therapy against amyloid formation (tafamidis that binds and stabilises the folded tetramer of transthyretin) has reached the market to date [45]. Some of the methods used to screen for small molecules able to modulate amyloid formation *in vitro* and in cellular assays are shown in Fig. 2. Each is complimentary with advantages and disadvantages, and progress requires application of several of these approaches in parallel as highlighted in Fig. 1.

### Accelerating amyloid formation of IDPs

**Fig. 3:**
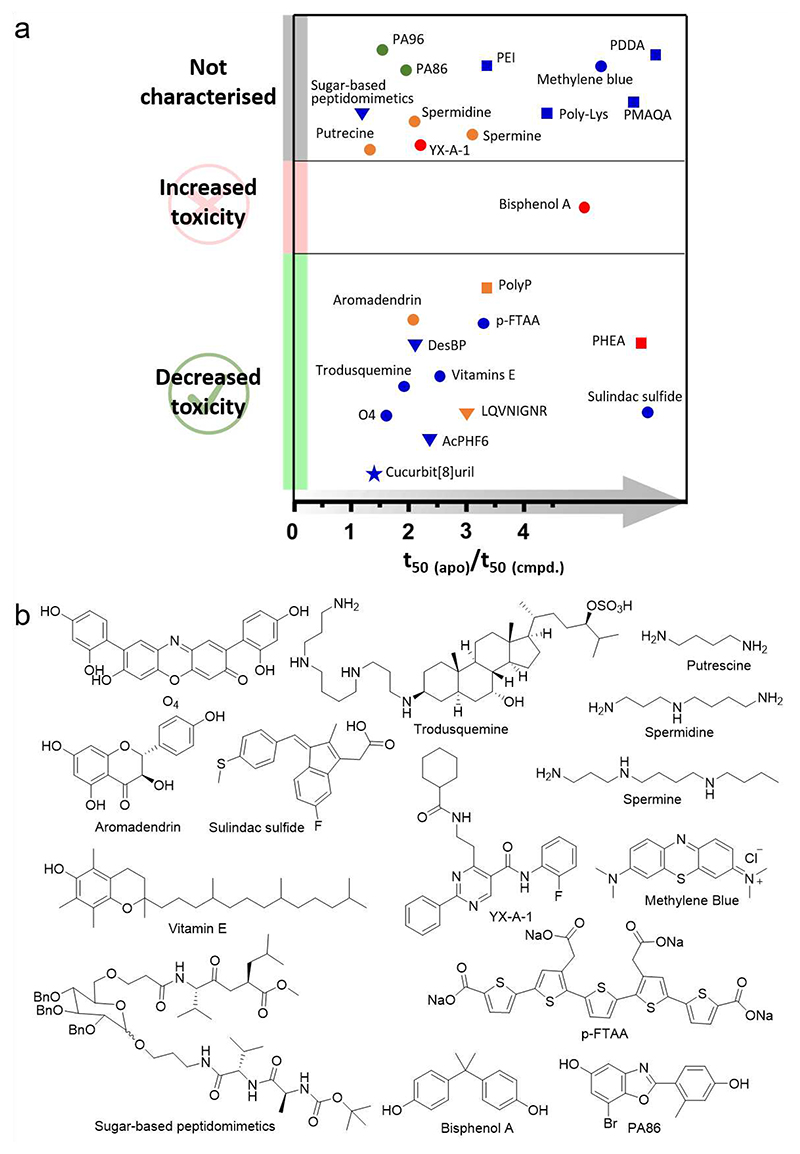
Accelerators of IDPs. (a) Relative t_50_ of the accelerators and their effect on IDP-induced cytotoxicity. Blue, red, green and orange represent Aβ, hIAPP, α-Syn and multiple targets, respectively. Circle, triangle, square and star represent small molecules, peptides, polymers and macrocycle, respectively. Of note, there are still a large number of accelerators whose effect on IDP-mediated cytotoxicity are not available. It would be interesting to determine the effect of these molecules, which might help to build a greater in-depth view of this possible therapeutic strategy. (b) Chemical structures of small-molecule accelerators discussed in this review.

Since the oligomeric species of amyloid assembly are thought to be primarily responsible for amyloid-associated cytotoxicity [21], one approach to treat amyloid would be to decrease their population by shortening their lifetime [16, 32, 50, 51]. One way of achieving this is to accelerate the conversion of oligomers into the stable cross-β amyloid fold. Protein modulators able to accelerate amyloid formation are scarce, with the chaperone SERF being one of very few examples of this kind [52]. Interestingly, SERF is itself an IDP, and has been shown to accelerate the aggregation of Aβ and α-Syn into amyloid (the latter an IDP associated with PD) [52]. Small-molecule accelerators of amyloid formation are also scarce compared with their inhibitory counterparts, but there have been some successes, as shown in Fig. 3 and listed in Table 1.

One mode-of-action by which small molecules can accelerate amyloid formation is by recruiting monomers or oligomers non-specifically and enhancing the nucleation rate of amyloid formation by increasing the local concentration of the protein/peptide precursors. Small molecules which form colloidal particles [53] or micelles [54, 55] have such a mechanism of action, although their general lack of specificity rules them out for development of therapeutics [56, 57]. Highly charged polymers, such as positively charged polyamino acids have also been shown to be effective amyloid accelerators of Aβ and other proteins, by interacting with their target(s) in a non-specific manner [54, 58–60]. In some cases a degree of specificity can still be achieved. For example, colloids formed by sulindac sulfide bind Aβ monomers non-specifically; while sulindac sulfide monomers in solution, or in equilibrium with their colloidal form, bind to hydrophobic cavities (in the vicinity of Gly33) in the Aβ fibril core specifically [53, 61].

Small-molecule accelerators of amyloid formation have also been discovered that bind to oligomeric precursors of amyloid formation [32, 50, 62]. For example, the orcein-related small molecule, O4, binds to hydrophobic residues (^12^VHHQKLVFFA^21^ and ^24^VGSNKGAIIG^33^) within Aβ_42_ oligomers [62]. The compound YX-A-1 was recently identified as a potent accelerator of amyloid formation of hIAPP (but not Aβ), enhancing fibril formation substoichiometric concentrations by binding oligomers in the lag phase of assembly [32]. The luminescent oligothiophene, p-FTAA, also binds to oligomers of Aβ (and prion protein [63]), and induces formation of β-sheet structures [64]. These studies highlight the different structural features of oligomers formed from different amyloid precursors or even from different microscopic steps of the sample precursor, which presumably rationalises their specificity.

As shown in Fig. 3, most known accelerators of amyloid formation show a protective effect on IDP-induced cytotoxicity, regardless of their chemical structure, mode-of-action or specificity [50, 65, 66]. Some have a dual benefit. For example, trodusquemine enhances the aggregation of Aβ_42_ and at the same time reduces the binding affinity of Aβ_42_ oligomers to cellular membranes, leading to an amelioration of toxicity in neuroblastoma cells [50]. These findings reinforce the notion that driving the formation of inert fibrils from cytotoxic oligomers could be a beneficial strategy for treating amyloid disease. However, this is not always the case. For example, bisphenol A (BPA) was identified as an effective promotor of hIAPP amyloid assembly, however, it failed to alleviate hIAPP-induced cytotoxicity in INS-1 cells [67]. Instead, BPA promotes the formation of hIAPP oligomers with enhanced toxicity and an enhanced ability to disrupt membranes [67]. This is still a valuable outcome in the quest to better understand the structure-function relationship of oligomers. Interestingly, while BPA failed to protect against hIAPP-induced cytotoxicity, derivatives of BPA resulted in inhibitors against hIAPP aggregation [68]. This highlights the fine balance of interactions that control amyloid assembly, with small changes in chemistry able to switch between accelerators and inhibitors by fine-tuning the shape of the aggregation energy landscape.

Some modulators show more complex behaviours on the amyloid formation of IDPs, with their effect depending on the target species, the concentrations of the modulator, and/or the assay conditions employed. For example, trodusquemine enhances the aggregation of Aβ_42_ [50], but inhibits the aggregation of α-Syn and suppresses α-Syn-induced cytotoxicity in neuronal cells [69]. Some modulators show different effects on the same target, depending on the concentration used in the assay. For example, trehalose inhibits hIAPP aggregation at low concentration, but promotes fibrillation of hIAPP at high concentrations [70]. This different behaviour of the same modulator at various concentrations could result from the ligand altering its own self-association at different concentrations (e.g. colloidal behaviour). Hence, it is crucial to assess the solubility and aggregation propensity of the small molecules themselves before drawing conclusions as to their mode-of-action.

### Inhibiting the assembly of IDPs into amyloid

The development of inhibitors of amyloidogenic proteins/peptides as therapeutic agents against protein-misfolding diseases has received much interest over the last decade [16, 18, 51]. The desired outcome of these inhibitors is to retard/block amyloid formation and prevent/reduce the formation of cytotoxic species. Due to the complexity of aggregation pathways, there are potentially many species to target, each of which can change the rate or outcome of assembly: monomeric precursors, oligomers and fibrils. Many different types of small molecules have been designed or identified to target these species, including small molecules, peptides, polymers, and nanoparticles. These inhibitors have been comprehensively reviewed recently by several groups [16, 18, 51, 78–81]. Here we focus on the recent development of small-molecule inhibitors of Aβ and hIAPP, using these systems as exemplars of the challenges and successes in this buoyant field. Several examples are listed in Table 2, and each is discussed below, grouped by the precursor(s) in amyloid formation that they target.

Discovering small molecules that bind to monomeric IDP precursors of amyloid formation is challenging, because such proteins are dynamically disordered and continually ‘on the move’. Nonetheless, various studies have shown that it is possible to specifically target these species and to inhibit amyloid formation, with successes resulting from combinations of *in vitro* screens and in-cell assays [32, 82–85]. For example, 10074-G5 which binds to intrinsically disordered c-Myc monomer is able to retard the primary and secondary nucleation pathways of Aβ_42_ by interacting with the monomeric peptides [82]. This type of interaction leads to a decrease in hydrophobicity and an increase of conformational entropy of Aβ_42_, which demonstrates the therapeutic possibility for the treatment of protein-misfolding diseases through an ‘entropic expansion’ mechanism [82]. The strategy of targeting monomers of IDPs has also been applied to hIAPP. YX-I-1 was identified as a potent inhibitor against amyloid formation of hIAPP via a combinatorial approach of nESI-MS and ThT bioassays [32]. It can specifically delay primary nucleation, secondary nucleation and elongation by binding to hIAPP monomers and further 2D NMR studies show that the inhibitor mainly interacts with residues in the regions 10-14, 17-20, 23-28 and the C-terminal Tyr37 [32]. Collectively, these studies demonstrate that it is possible to specifically target monomeric IDPs and control their assembly into amyloid.

Successes in targeting oligomers in Aβ/hIAPP amyloid assembly have also been reported. For example, CurDAc, a water-soluble curcumin derivative, induces the formation of hIAPP oligomers and these species reduce RIN-5F cell viability [86]. Via a drug-repurposing strategy, cloridarol (used for the treatment of cardiovascular disease) was identified as an effective inhibitor of hIAPP fibrillation and reduces the hIAPP-induced cytotoxicity in RIN-m5F cells [87]. Molecular Dynamics (MD) simulations revealed that cloridarol preferentially binds to C-terminal β-sheet region of hIAPP oligomers via a combination of hydrophobic interactions, hydrogen bonding and π-π stacking [87]. The different effects of the inhibitors on hIAPP-induced cytotoxicity highlight that inhibition of aggregation does not necessarily correlate with reducing cytotoxicity.

Finally, targeting fibrils themselves with small molecules has been achieved, and could be an effective strategy, assuming that fibrils represent the inert end products of amyloid assembly (which is not necessarily the case, especially given their role in disease transmission, seeding and secondary nucleation that catalyses oligomer and amyloid formation). The near-atomic resolution structures of fibrils generated from pure proteins/peptides *in vitro* or from patient samples provide a structural basis to specifically target these assembly end products. Most of these type of inhibitors are peptide-based [41, 42]. In some cases, binding to amyloid fibrils causes their disassembly [86, 88–91]. The resulting conversion of fibrils into soluble oligomers can result in the generation of non-toxic [89, 90] or toxic species [86]. These molecules are important chemical tools to stabilise the transient and dynamic oligomeric species for more detailed molecular studies. The different toxicity profiles of the oligomers again highlight targeting aggregation and modulating cytotoxicity should be considered as independent events.

### Characterising the interactions between IDPs and small-molecule modulators

**Fig. 4:**
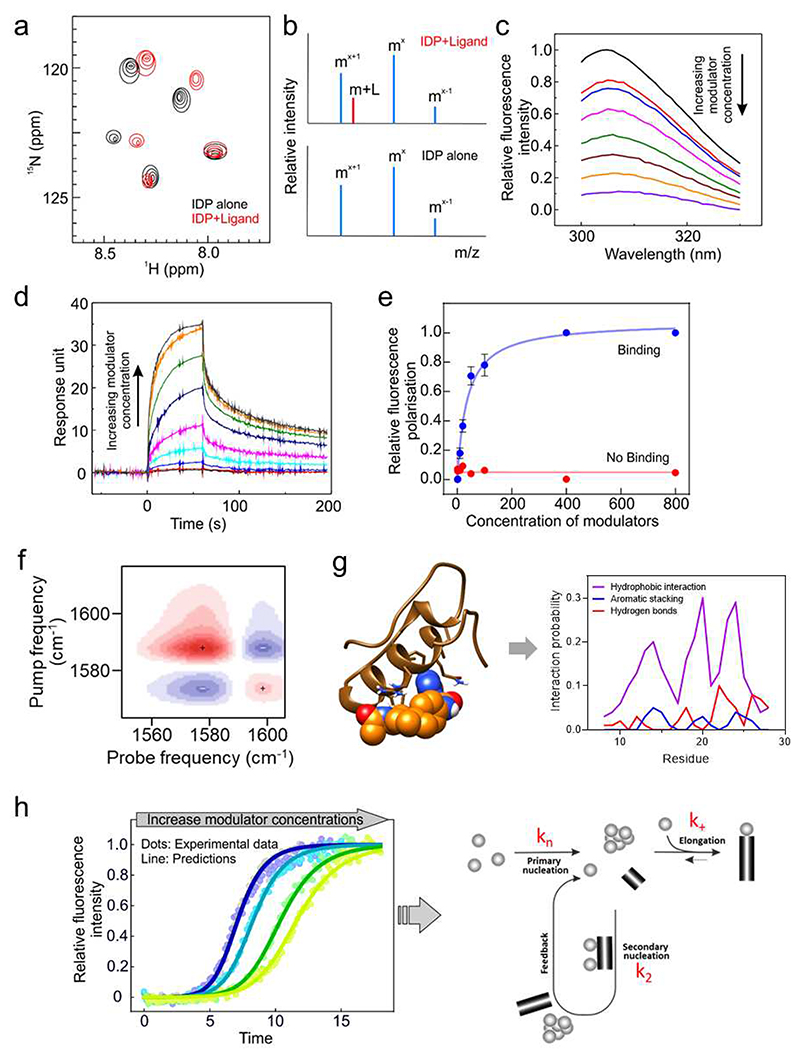
Biochemical and biophysical approaches used for the characterisation of the interaction between IDPs and small molecules. (a) Representative 2D NMR spectrum of an IDP with (red) and without (black) a small molecule. (b) nESI mass spectra show the mass addition of a ligand to the target, suggesting the interaction between the target and the ligand. (c) Fluorescence quenching caused by ligand binding. Stern-Volmer analysis of the fluorescence titration data enables extraction of the K_d_. These data were taken from ref [32]. (d) SPR measurement showing ligand binding (K_d_) as well as the association (*k*_on_) and dissociation (*k*_off_) rate constants. These data were taken from ref [32]. (e) Fluorescence polarisation (FP) can provide indications of binding/no binding of ligands. Fitting the FP titration can yield the binding affinity of the ligand. (f) 2D FTIR can provide binding site information of the inhibitor at residue-level resolution. (g) MD simulations can provide detailed descriptions of the conformational ensembles of IDPs and specific information about the IDP-ligand interactions at an atomic-level. MD simulation can predict the favoured interactions formed between IDP and ligand, such as hydrogen bonding, aromatic interaction, charge-charge interaction, etc. (see [29]). (h) Measurement of amyloid formation using ThT fluorescence can be used to determine the mechanism of amyloid formation and the species that bind ligands that affect the rate of assembly into amyloid. These data were taken from ref [32].

Identifying the targets of small-molecule modulators and probing their mode-of-action have been revolutionised in recent years by the application of kinetic analyses that are able to define the step(s) in amyloid formation that are affected by the addition of a specific ligand [104]. The online programme AmyloFit has been widely applied to study the microscopic steps in amyloid assembly for many proteins, including the Aβ [105, 106] and hIAPP [32, 107], and used to define the targets and mechanism(s) of action of modulators of amyloid assembly [32, 92, 104]. One of the major challenges remaining is to map the binding site of the ligands, so as to understand their mechanism of action in structural/molecular detail. For IDPs it is difficult to answer this critical question because of the dynamics and complexity of the system. As discussed above, most interactions between IDPs and small molecules are weak (∼ μM), involving rapid on/off rates to the protein, which itself is a dynamic moving target [29, 82, 108]. These features make it difficult to detect or quantitatively measure the binding affinity of the modulators and to map their binding site(s). Significant progress has been made recently in both experimental and *in silico* approaches. These are summarised in Fig. 4. Amongst these, ligand and protein-detected NMR methods, especially at high field strengths (e.g. 950 MHz) are ideal for measuring ligand binding to an IDP and for mapping residues involved in single atom detail [32, 101, 109] (Fig. 4a). nESI-MS coupled with ion mobility-MS (IM-MS) enables discrimination between non-specific and specific binders [33], and can reveal the effect of binding on the conformational distribution of the IDP [110] (Fig. 4b). Methods such as surface plasmon resonance (SPR), fluorescence polarisation (FP) and bio-layer interferometry (BLI) provide methods of determining affinity, with fluorescence titration using Stern-Volmer analysis being particularly fruitful for low affinity measurements [32, 82, 109] (Fig. 4c-e). SPR and BLI can provide information about the association (*k*_on_) and dissociation (*k*_off_) rate constants of the protein-ligand interactions. By varying the experimental temperature (e.g. using fluorescence titrations), thermodynamic parameters such as the entropy change (ΔS°) and enthalpy change (ΔH°) can be obtained [111]. Two-dimensional infrared spectroscopy (2D FTIR) using isotope-labelled protein can be used to map binding sites of the inhibitor with residue-level resolution [112] (Fig. 4f), while large scale calculations using supercomputers, guided by experimental restraints, can be used to describe the dynamic ensemble of ligand bound states [29]. Linked with parallel kinetic analysis of the mechanism of action of the modulator (Fig. 4g,h), the tools are now in place for a full description of the mode-of-action of the modulators of amyloid formation and how their implementation can tip the energy landscape to favour or disfavour aggregation or even to change its course towards different products of amyloid assembly.

## Conclusions and outlook

In recent years, substantial progress has also been made in understanding of the kinetic mechanisms of amyloid formation and the structure of the ultimate amyloid fibrils formed [3, 113]. Significant progress has been made in the development of small-molecule modulators towards amyloid-forming proteins/peptides [16, 79, 80]. Here we have reviewed current approaches that are being used in the search for small molecules that are able to modulate the self-association of IDPs into amyloid, and discuss recently reported inhibitors and accelerators of amyloidogenic proteins/polypeptides, focusing on hIAPP and Aβ. We also discuss the advances made in probing small-molecule-IDP interactions by biochemical and biophysical approaches, both experimental and computational. With the development of these methods, it is now possible to detect weak binding of small molecules to IDPs, identify the potential targets of these molecules in IDP-associated amyloid formation, and investigate their mode-of-action in detail. The stage is thus set for an optimistic future that these weak binding molecules may cast new light on how and why IDPs aggregate into amyloid fibrils of different structure specific to different diseases [3, 13]. They also reveal how small changes in the population of different amyloid precursors brought about by weak binding of ligands can fundamentally change the outcomes of amyloid assembly, and can be used to determine the structure-function relationships of the transient and dynamic intermediates of amyloid assembly.

Many questions and challenges in this exciting field remain. Since protein-misfolding diseases are under kinetic control, modulating the aggregation kinetics by altering the rates of different microscopic steps could be an effective way to develop possible therapeutic agents. One fundamental question remaining, however, is whether it is better to slow down or speed up aggregation. The answer to this question will require a better understanding of disease-causing mechanisms, specifically which species in the aggregation energy landscape are cytotoxic and which are benign. It is important to note that cross-seeding of different amyloid protein precursors has been observed and also some proteins are known to aggregate in the same disease (e.g. Dementia with Lewy bodies (DLB) [114], AD, and T2D [10, 11]). A crucial question is whether it is better to develop broad-spectrum modulators able to target multiple protein precursors, e.g. Aβ and α-Syn, Aβ and Tau or Aβ and hIAPP (in LB, AD and T2D, respectively), rather than hunting for small molecules with high specificity. Since the cellular environment is more complex than the experimental conditions of *in vitro* experiments (such as the presence of metal ions, membranes, molecular chaperones and/or crowding) [115], a better understanding of how modulators behave under physiological conditions is crucial for the transition of small molecules discovered *in vitro* or in cell lines into effective therapeutic agents. Another question under hot debate is whether it is possible to identify modulators to IDPs with high binding affinity (i.e. sub-μM) and whether tight binding is required for the effectiveness and specificity required for any small molecule in the clinic. Encouragingly, several examples have now been reported of the successful development of modulators which alter the aggregation kinetics of IDPs via weak and dynamical binding to their targets [29, 32, 82]. The physicochemical properties of the modulators needs also to be considered, such as their stability, specificity, solubility, cell permeability, ability to pass through the blood brain barrier (for amyloidosis causing neurodegeneration) and their own aggregation propensity, before conducting further functional studies.

Apart from the reversible modulators of amyloid formation discussed in this review, other promising strategies in term of modulator design have been reported. One of them is the development of covalent modulators which have high potency [116–118]. Another promising strategy is the application of ‘molecular glue’ or proteolysis-targeting chimera (PROTAC), as strategies to target and degrade amyloid precursors. Li and colleagues identified molecules which interact with both huntingtin (mHTT) (the causative agent of Huntington’s disease) and autophagosome protein microtubule-associated protein 1A/1B light chain 3 (LC3) [119]. They showed that the molecules can specifically target mHTT to autophagosomes, reduce the mHTT levels, and rescue disease-relevant phenotypes in cells and *in vivo* in fly and mouse models of Huntington’s disease [119]. Such approaches could be powerfully applied synergistically with small-molecule modulators of amyloid formation, such that the course of aggregation can be controlled by small switches in the concentration of relevant misfolded precursor states. Since metal ions can play important roles in amyloid formation (such as metal ion-induced Aβ aggregation), bifunctional modulators have been identified that synergistically chelate metal ions and inhibit aggregation into amyloid [120, 121].

In conclusion, despite its long and sometimes tortuous history [1, 2, 113], the amyloid field is currently full of excitement and hope, with the enhancement in our fundamental understanding of aggregation mechanisms and the array of new strategies and small molecules able to control aggregation laying strong foundations for the much-needed breakthroughs in the treatment of amyloid diseases in the years ahead.

## Figures and Tables

**Table 1:** Accelerators of amyloid-formation of different proteins/peptides. List of small-molecule accelerators that have been development for various amyloid-forming proteins/peptides. They are classified based on their chemical structures, and their mode-of-action and effect on IDP-induced toxicity are summarised.

Name	Chemical structure	Amyloid precursor	Mechanism of action	Toxicity profile	Ref
O4	Small molecule	Aβ_42_	O4 binds to hydrophobic amino acid residues (^12^VHHQKLVFFA^21^ and ^24^VGSNKGAIIG^33^) of Aβ_42_ and stabilises β-sheet rich species.	Reduce cytotoxicity	[62]
Bisphenol A (BPA)	Small molecule	hIAPP	BPA facilitates the secondary structure transition of hIAPP and induces formation of oligomeric species.	Increase hIAPP- induced membrane disruption and hIAPP-induced INS-1 cell apoptosis.	[67]
PA86	Small molecule	α-Syn	PA86 increases the elongation growth rate of α-syn. PA86 forms no direct interactions with α-Syn monomer.	N.D.	[46]
Aromadendrin	Small molecule	hIAPP and Aβ_42_	Aromadendrin promotes the structural conversion of species in the lag and early growth phases.	Reduce Aβ- and hIAPP-induced toxicity	[66]
Trodusquemine	Small molecule	Aβ_42_	Trodusquemine increases the rate of surface-catalysed secondary nucleation.	Inhibit binding of oligomers to neuroblastoma cells. Increase the number of aggregates in *C. elegans* models, but reduce Aβ_42_-induced toxicity.	[50]
YX-A-1	Small molecule	hIAPP	YX-A-1 interacts predominantly with oligomers formed in the lag phase.	N.D.	[32]
Vitamin E	Small molecule	Aβ_42_	Vitamin E forms micelles to promote Aβ_42_ aggregation.	Improve the fitness of AD *C. elegans* model.	[55]
Polyamines (spermine, spermidine, and putrescine)	Small molecule	Aβ_40_, α- Syn	All three polyamines bind to the monomeric Aβ_40_ around residues 4-5, 15-17 and 27-28.	N.D.	[71] [72]
Sugar-based peptidomimetics	Small molecule	Aβ_42_	The peptides interact with Aβ_42_ probably via p-stacking.	N.D.	[73]
Sulindac sulfide	Small molecule	Aβ_40_	Sulindac sulfide forms colloidal particles which recruits monomers and increase local peptide concentration.	Reduced Aβ_42_-induced toxicity.	[53]
p-FTAA	Small molecule	Aβ_42_	p-FTAA bound to Aβ oligomers formed at the early stage of aggregation and induced formation of β-sheet structures.	Reduce Aβ_42_-mediated cytotoxicity	[64]
Methylene Blue	Small molecule	Aβ_42_	Methylene blue promotes both filament nucleation and elongation.	N.D.	[74]
PDDA, PEI and Poly-Lys	Polymer	Aβ_42_	PDDA increases the local concentration of Aβ_42_ and decrease self-repulsion of Aβ_42_.	N.D.	[58]
Polyphosphate (PolyP)	Polymer	Aβ_42_, α-Syn, Tau, CsgA	It is speculated that PolyP binds to monomers and increases the local concentration. It also accelerates biofilm formation (a functional amyloid) in bacteria (curli formation of CsgA).	Reduce α-Syn and Aβ_42_ induced toxicity in cells and *C. elegans* models.	[59]
Poly(2-hydroxyethyl acrylate) (PHEA)	Star polymers	hIAPP	PHEA interacts with N-terminal residues of hIAPP and increases the local peptide concentration.	Reduce hIAPP-induced toxicity both *in vitro* and *ex vivo.*	[75]
Polymethacrylat e-copolymer (PMAQA)	Polymer	Aβ_40_	PMAQA induces a β-hairpin structure by binding to regions spanning Lys16-Val24 and Ala30-Val40.	N.D.	[60]
DesBP	Bicyclic peptide	Aβ_42_	DesBP interacts weakly with the monomeric Aβ_42_ and increases both primary and secondary nucleation are, but not elongation.	Reduce Aβ_42_-induced toxicity in *C. elegans*models.	[65]
LQVNIGNR	Peptide	Ure2, Tau- N244- F378, α- Syn	This peptide forms vesicular assemblies which promotes conformational transitions of oligomers.	Decrease oligomer-induced membrane leakage.	[54]
AcPHF6	peptide	Aβ_40_, Aβ_42_	The peptide is likely to interact with Aβ monomer.	Reduce Aβ_40_ and Aβ_42_ mediated toxicity in neuronal cells.	[76]
Cucurbit[8]uril	Macrocycle	Aβ_42_	It preferentially targets Phe residues in Aβ_42_ and increases the size of the Aβ_42_ aggregates.	Reduce Aβ_42_-induced toxicity in neuronal cell line.	[77]

**Table 2:** Recently-reported small-molecule inhibitors of Aβ and hIAPP. Small-molecule inhibitors against Aβ and hIAPP are listed, and their mode-of-action and effect on IDP-induced toxicity are summarised.

Name	Amyloid precursor	Mechanism	Toxicity profile	Ref
Bexarotene	Aβ_42_	Bexarotene selectively targets the primary nucleation step in Aβ_42_ aggregation.	Reduce the Aβ_42_-induced toxicity in neuroblastoma cells and Aβ_42_ *C. elegans*model.	[92]
Rhodanine-based compounds	Aβ_42_	These molecules can delay oligomer formation and reduce the overall oligomer production.	N.D.	[93]
Polyphenolic biflavonoids	Aβ_42_	These molecules inhibit Aβ_42_ fibrillation and disassemble preformed Aβ_42_ fibrils. SAR study identified an essential role of the hydroxyl groups on the molecules.	N.D.	[88]
10074-G5	Aβ_42_	10074-G5 retards primary and secondary nucleation pathways by binding to monomeric Aβ_42_.	Rescue a *C. elegans* model of Aβ-associated toxicity.	[82]
Anle138b	Aβ_42_	Anle138b prevents Aβ_42_ oligomers induced pore formation in membranes.	Ameliorate hippocampal synaptic, spatial reference memory, and transcriptional homeostasis in AD mouse model.	[94]
Vitamin A (retinoic acid)	Aβ_42_	Vitamin A inhibits the primary and secondary processes of Aβ_42_ fibrillation.	Vitamin A reduces Aβ_42_ aggregates and increases the total fitness of *C. elegans.*	[55]
Catechol-containing isoflavone	Aβ_42_ and Tau	Several derivatives inhibit the aggregation of Aβ_42_ and Tau.	Reduce the Aβ plaques in the brain and improves the memory deficits in AD mice model.	[95]
Genistein	Aβ_42_ and hIAPP	Genistein inhibits the aggregation of hIAPP and Aβ_42_ by preventing the conformational transition of peptide monomers into P-sheet structures.	Prevent hIAPP (RIN-m5F) or Aβ_42_-induced (SH-SY5Y) cytotoxicity possible thorough reducing peptide-induce membrane leakage.	[84]
AQ-4, THQ-1, DHQ-1, DHQ-2 and BF-3	Aβ_40_	Compounds were identified through a high-throughput screening in the presence of membrane.	N.D.	[31]
BIBA	Aβ_40_	BIBA inhibits the aggregation of Aβ_40_. BIBA interacts weakly with peptide monomers. Docking studies show that BIBA interacts with seven residues which are Glu22, Phe19, Val18, Lys16, Gln15, His6 and Arg5.	Inhibit Aβ-induced paralysis of *C. elegans* and reduce Aβ plaques in the brain of AD mice model.	[96]
Anle145c	hIAPP	Anle145c thermodynamically traps hIAPP in non-cytotoxic oligomers and converts hIAPP amyloid fibril into non-toxic oligomers.	Reduces hIAPP-induced toxicity in INS-1E cells and human MJS cells.	[89]
Silybins	hIAPP	Stereospecific inhibition of hIAPP aggregation was observed. MD simulations show that Silybin B interacts with the Ser20-Ser29, His18, the N-terminal domain, and Asn35.	Protect INS-1 cells from hIAPP toxicity more efficiently than silybin A.	[97]
Cloridarol	hIAPP	Cloridarol reduces the overall quantity of amyloid fibrils. MD revealed that it binds to C-terminal β-sheet region of hIAPP oligomers.	Protect islet β-cells from hIAPP-induced cytotoxicity.	[87]
Yakuchinone B derivatives	hIAPP	Molecular docking shows that molecules interact with hIAPP monomer through hydrogen bonding and hydrophobic interactions.	Reduce IAPP-induced toxicity for BRIN-BD11 cells.	[98]
2- phenylbenzofurans	hIAPP	2-Phenylbenzofurans prevent the fibril formation of hIAPP.	N.D.	[99]
YX-I-1	hIAPP	YX-I-1 inhibits primary nucleation, secondary nucleation and elongation of hIAPP fibrillation by interacting with peptide monomers.	N.D.	[32]
Tetracycline derivatives	hIAPP and Aβ	Tetracyclines inhibit the fibril formation of hIAPP and Aβ. They can also effectively disaggregate matured fibrils. MD simulations were carried to study the peptide-small molecule interactions.	Rescue peptide-induced cytotoxicity for INS-1 and SH-SY5Y cell lines.	[90]
Tryptophan-galactosylamine conjugates	hIAPP and Aβ_42_	These conjugates inhibit aggregation of hIAPP and Aβ_42_ and disassemble pre-formed hIAPP and Aβ_42_ fibrils.	Reduce the cytotoxicity induced by Aβ_42_ (SH-SY5Y) and hIAPP (HEK-293).	[91]
Lithospermic acid (LA)	hIAPP	LA inhibits hIAPP aggregation by binding to hIAPP monomers. Docking studies show that LA interacts with Arg11, Asn14, Phe15, Asn21, Ala25, Ile26 and Val32.	Reduce hIAPP-induced cytotoxicity of INS-1 cells.	[83]
Naphthoquinone-based hybrids	PHF6, hIAPP and Aβ_42_	These molecules inhibit the fibril formation of all the three peptides and disrupt the matured fibrils. Molecular docking shows interaction between small molecules and the monomeric peptides.	Reduce the cytotoxicity induced by PHF6 (SH-SY5Y), Aβ_42_ (SH-SY5Y) and hIAPP (HEK-293).	[100]
DM1	hIAPP	It interacts with the monomeric peptide by stabilising and/or perturbing the helix conformation at the N-terminus. DM1 is likely to interact strongly with positively charged residues (Lys1 and Arg11) and hydrophobic domain of the peptide (Leu12-Val17).	Reduce hIAPP-induced cytotoxicity in RIN-m cells.	[101]
CurDAc	hIAPP	CurDAc inhibits hIAPP aggregation and disassembles hIAPP fibrils. CurDAc promotes the formation of oligomers. NMR studies show that CurDAc interacts residues T4, C7, A8, Q10-N14, L16-S19, I26 and S29.	Increase hIAPP-induced cytotoxicity in RIN-5F cells.	[86]
Resveratrol derivatives (4’- DMPR and 4’-O-PR)	hIAPP	Both molecules abolished hIAPP amyloid growth and protected hIAPP-induced membrane damage.	N.D.	[102]
DP-128	Pan amyloid inhibitor	DP-128 inhibits the aggregation of a number of different amyloidogenic proteins, including hIAPP, Aβ_40_ and Aβ_42_.	N.D.	[103]
HUP7TH	Pan amyloid inhibitor	HUP7TH inhibits the aggregation of a number of different amyloidogenic proteins, including hIAPP, Aβ_40_ and Aβ_42_.	N.D.	[103]

## Key summary points

– Protein-misfolding diseases are defined as kinetic diseases which are modulated by different microscopic events with defined rate constants.
– Small-molecule modulators towards IDPs are powerful chemical tools for the investigation of amyloid formation mechanisms and probing molecular mechanisms of amyloid-induced cytotoxicity.
– Targeting amyloid aggregation and modulating IDP-induced cytotoxicity should be considered as two independent events.
– Both inhibitors and accelerators of IDPs are equally important and can inform whether it is better to slow-down or speed-up the self-assembly of IDPs into amyloid.

## Abbreviations

Aβ: Amyloid β
AD: Alzheimer’s disease
ALS: Amyotrophic Lateral Sclerosis
BiFC: bimolecular fluorescence complementary assay
BLI: bio-layer interferometry
BPA: bisphenol A
cryo-EM: cryo-electron microscopy
2D FTIR: two-dimensional infrared spectroscopy
FP: fluorescence polarisation
hIAPP: human islet amyloid peptide
HTS: high-throughput screening
IDPs: Intrinsically disordered proteins/peptides
IM: ion mobility
LA: lithospermic acid
MD: molecular dynamics
nESI-MS: native electrospray ionisation mass spectrometry
PD: Parkinson’s disease
PolyP: polyphosphate
PMAQA: polymethacrylate-copolymer
PROTAC: proteolysis-targeting chimera
T2D: type II diabetes
ThT: Thioflavin T
αSyn: α-Synuclein
SynAggreg: Synergistic Aggregation Modulator Assay
SPR: surface plasmon resonance.
